# Presence of a pre-hospital enhanced care team reduces on scene time and improves triage compliance for stab trauma

**DOI:** 10.1186/s13049-019-0661-z

**Published:** 2019-09-06

**Authors:** Alan Cowley, Mark Durham, Duncan Aldred, Richard Crabb, Paul Crouch, Adam Heywood, Andy McBride, Julia Williams, Richard Lyon

**Affiliations:** 1 South East Coast Ambulance Service NHS Foundation Trust (SECAmb), Nexus House, 4 Gatwick Road, Crawley, RH10 9BG UK; 2 Air Ambulance Kent Surrey Sussex, Rochester Airport, Maidstone Road, Chathan, Rochester, ME5 9SD UK; 3 University of Surrey, Stag Hill, Guildford, GU2 7XH UK

**Keywords:** Paramedic, Prehospital, Trauma, Critical care, Stabbing, Scene time

## Abstract

**Background:**

A reduction in pre-hospital scene time for patients with penetrating trauma is associated with reduced mortality, when combined with appropriate hospital triage. This study investigated the relationship between presence of pre-hospital enhanced care teams (ECT) (Critical Care Paramedics (CCPS) or Helicopter Emergency Medical Service (HEMS)), on the scene time and triage compliance, of penetrating trauma patients in a UK ambulance service. The primary outcome was whether scene time reduces when an ECT is present. A secondary outcome was whether the presence of an ECT improved compliance with the trust’s Major Trauma Decision Tree (MTDT).

**Methods:**

All suspected penetrating trauma incidents involving a patient’s torso were identified from the Trust’s computer-aided dispatch (CAD) system between 31st March 2017 and 1st April 2018. Only patients who sustained central penetrating trauma were included. Any incidents involving firearms were excluded due to the prolonged times that can be involved when waiting for specialist police units. Data relevant to scene time for each eligible incident were retrieved, along with the presence or absence of an ECT. The results were analysed to identify trends in the scene times and compliance with the MTDT.

**Results:**

One hundred seventy-one patients met the inclusion criteria, with 165 having complete data. The presence of an ECT improved the median on-scene time in central stabbing by 38% (29m50s vs. 19m0s, *p* = 0.03). The compliance with the trust’s MTDT increased dramatically when an ECT is present (81% vs. 37%, odds ratio 7.59, 95% CI, 3.70–15.37, *p* < 0.0001).

**Conclusions:**

The presence of an ECT at a central stabbing incident significantly improved the scene time and triage compliance with a MTDT. Ambulance services should consider routine activation of ECTs to such incidents, with subsequent service evaluation to monitor patient outcomes. Ambulance services should continue to strive to reduce scene times in the context of central penetrating trauma.

## Background

Trauma is the leading cause of death in patients under the age of 40 and approximately five million people worldwide die each year as a result of traumatic injury [1]. Injuries from knife crime are on the increase in the UK [2]. As the initial point of contact for many of these patients, emergency medical services can play a pivotal role in the mortality and morbidity seen in this group of patients.

Penetrating trauma to the torso poses significant challenges to pre-hospital care providers, due to the non-compressible nature of the underlying anatomy. It has long been an acknowledged principle that such patients benefit from expeditious transport to a specialist trauma centre [3, 4]. The development of the ‘Golden Hour’ is a widely accepted doctrine attributed to R Adams Cowley [5] and later supported in various studies [6, 7]. Whilst it has not been without contention; a large prospective trial failed to find a correlation between pre-hospital intervals and in-hospital mortality [8], the general premise has nonetheless been widely accepted, and developed to include the concept of the “Platinum ten minutes”. This states that pre-hospital care teams treating major trauma patients (such as victims of penetrating central trauma), should aim to remain on scene for just ten minutes or less [9]. The exact origin of the ten minute figure is unclear, but the time criticality of such patients is undeniable, and has recently been shown clearly by a study looking at HEMS units in Texas [10].

The ECTs available within South East Coast Ambulance Service NHS Foundation Trust (SECAmb) are Critical Care Paramedics (CCPs) and a Helicopter Emergency Medical Service (HEMS). The geographical area covers roughly 3600 mile^2^, and includes urban, rural and semirural areas. The area has a resident population of 4.5 million, with a transient population up to 8 million. In depth demographic data can be found on the trust’s website [11]. The region is covered by ten CCP teams distributed across the trust’s geographical area. Each geographical team comprises seven CCPs, including two Practice Leads. SECAmb’s CCPs are paramedics who have a minimum 3 years post-registration experience and have completed a post graduate certificate as a minimum, although a majority have completed or are working towards an MSc in advanced practice. They specialise in all high acuity care, including trauma. There is one HEMS unit, with one doctor-paramedic team operating 24 h per day and another team between the hours of 0700–0000. They are tasked predominantly (though not exclusively) to trauma. Both HEMS and CCPs have specialist training beyond that of frontline ambulance crews (typically staffed by a combination of paramedics, technicians, or support staff). As well as this, both forms of ECT regularly practice high acuity incidents, and also discuss decision-making and Crew Resource Management (CRM – a formalised appraisal of human, technical and environmental factors surrounding team function) in recent incidents, by way of governance through reflection.

The ECTs are tasked separately from each other, but both by dedicated dispatch desks who work collaboratively on the majority of incidents. A CCP will be allocated to any report of a central stabbing, provided they are available. The HEMS tasking follows a novel non-clinical system and is examined in more detail in Munro et al. [12]. As well as these criteria for automatic dispatch of a CCP, or HEMS, they can also be requested by ambulance crews who arrive on scene and feel they can add benefit, or an update is provided to this effect.

Few studies have looked into the effect of ECTs on the scene time and triage of central stabbing specifically. Eckstein & Alo [13] suggested that advanced training of paramedics can result in a shorter scene time, and several studies have attempted to look at the effect of a physician-led service (such as HEMS) on the scene times of major trauma [14, 15]. Frequency of exposure to trauma can also be related to clinicians’ confidence and accuracy in triage [16].

The premise underpinning these findings is that ECTs have advanced training and significantly greater exposure to central stabbing incidents, and so are well placed to ensure optimum patient management, which generally involves minimal intervention on scene and a time critical transfer to a MTC. Since the increased exposure is borne of selective tasking, and it is not feasible to provide this level of specialist training to all frontline paramedics, non-ECT personnel are reliant on their own continued professional development, annual ‘key skills’ refreshers and the basic training of the JRCALC guidelines [17]. This can mean that while JRCALC guidelines are quite clear in stating that in the context of a central stabbing, a “time critical transfer to a major trauma centre” should be undertaken, it may take a greater level of training and experience to translate this into the chaotic, dynamic, and often emotionally demanding scene of penetrating trauma [18, 19]. The study will examine the relationship between the presence of an ECT, and the total on-scene time, as well as and triage compliance.

This service evaluation aimed to use retrospective observational data to evaluate the impact of the presence of an enhanced care team (ECT) on the pre-hospital scene time in the context of penetrating trauma. The primary outcome was to evaluate whether scene times are reduced when an ECT is present. A secondary outcome was whether the presence of an ECT improved compliance with the trust’s Major Trauma Decision Tree (MTDT), which mandates that central stabbings be conveyed directly to one of the Major Trauma Centres (MTCs) within the trust’s geographical area.

## Methods

### Setting

SECAmb is an urban, suburban and rural NHS funded ambulance service that broadly encompasses the counties of Sussex, Surrey and Kent, and receives over 1,000,000 calls each year [20] It employs over 2000 clinical staff of which 60 were operating as CCPs as of 30th April 2017.

### Patient selection

Patients were identified using SECAmb’s Computer Aided Dispatch (CAD) system, which records incident details. The historical CAD was searched to include all incidents over a one-year period from the 1st April 2017 to the 31st March 2018. All incidents categorised as “stabbing” or “stabbing central” were included. The category assigned to the incident is chosen by the emergency call operator at the time of the call. It is acknowledged within this evaluation that if a stabbing incident was incorrectly categorised, then it could be missing from the dataset.

The CAD notes and the (automatically anonymised) patient report form were examined to ensure central penetrating trauma had occurred. For the purpose of the study, and to match with the MTDT, ‘central’ is defined as proximal to the knees and elbows. From the remaining incidents, the times of all resource arrival (single response vehicle, ambulance, CCP, HEMS) were noted, as well as the time at which the patient departed scene. The qualification of non-ECT resources (e.g. technician, paramedic etc) was irrelevant to the aims of this study, since clinical interventions were not examined. Any incidents involving firearms were excluded due to the prolonged times that are often involved when waiting for specialist firearms police units. In addition, the destination hospital of the patient was recorded.

### Statistical analysis

Data were collated using Microsoft Excel™. Average scene times (the time from the first ambulance response arriving on scene, to the time the patient left scene) were initially expressed using descriptive statistics (median, and interquartile range - IQR), and then any difference between the means of the two groups was tested for significance using an unpaired, two-tailed t-test using GraphPad online software (www.graphpad.com/quickcalcs/catMenu/).

Triage decisions (MTC or TU) were expressed initially using descriptive statistics (percentage), and the odds ratio calculated.

## Results

During the period reviewed, 171 patients met the inclusion criteria, and complete data were available for 165. Patients with partial data are included in the results where appropriate. Baseline characteristics are shown in Table 1.

**Table 1:** Baseline characteristics

Age in years (±SD)		33.5 (±16.2)
Gender	Male (%)	146 (85%)
	Female (%)	25 (15%)
Time of call (%)	Midnight-05:59	41 (24%)
	06:00–11:59	23 (14%)
	12:00–17:59	32 (19%)
	18:00–23:59	75 (44%)
Highest clinical response (%)	Non-ECT	72 (42%)
	CCP	67 (39%)
	HEMS	14 (8%)
	CCP and HEMS	17 (10%)
Median travel time to hospital (Interquartile range)mm:ss	TU	00:18:00 (06:45–28:00)
	MTC	00:31:30 (26:00–38:30)
Site of injury (%) (this includes incidents with more than one body area involved – “multiple” describes incidents where there is more than one stab wound, but the clinician has not recorded the sites separately)	Chest	51 (27%)
	Abdomen	49 (26%)
	Upper Leg	38 (20%)
	Back	20 (11%)
	Neck	12 (6%)
	Upper Arm	10 (5%)
	Buttock	5 (3%)
	“Multiple”	5 (3%)
	Head	1 (< 1%)

**Fig. 1:**
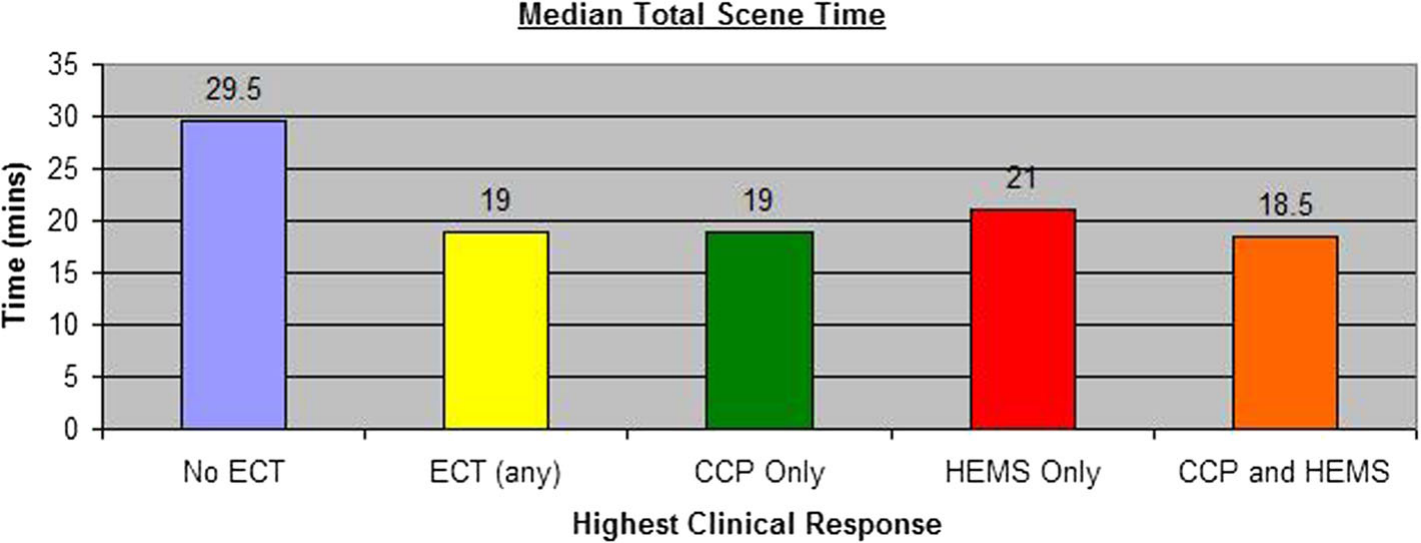
Median total scene time. Bar chart showing the median scene time, following arrival of the first conveying resource. The chart shows these times where there was no ECT present, any form of ECT and then by type of ECT. Median scene time is significantly longer in the absence of an ECT (*p* = 0.03)

Median total scene time for non-ECTs was 29 min and 30 s (29:30 – IQR 18:00–37:00). For ECTs overall this was 19 min (IQR 12–30 min), 19 min if a CCP was in attendance (IQR 12–30 min), 21 min if HEMS attended (IQR 10–34 min), and 18.5 min if both ECT units attended (IQR 13.75–32 min). This is illustrated in Fig. 1. An unpaired, two-tailed t-test was used to compare mean scene times for ECTs overall, with those of non-ECTs; the means here were 26:46 (SD 14:41), and 31:53 (SD 18:00) respectively. There was a significant difference between ECT (standard deviation 14.5 min) and non-ECT (standard deviation 18 min) groups; t (165) =2.14, *p* = 0.03, 95% CI, − 10.22—0.40.

Overall, 63% (*n* = 104) of patients travelled to an MTC, and 37% (*n* = 61) travelled to a TU. An ECT attended scene in 58% (*n* = 97) of incidents. When an ECT was in attendance, patients travelled to an MTC in 81% of cases (*n* = 79), and if no ECT was in attendance, the patient travelled to an MTC in 37% of cases (*n* = 25). Patients were therefore more likely to be triaged to an MTC by an ECT (odds ratio 7.59, 95% confidence interval, 3.70–15.37, *p* < 0.0001).

## Discussion

This service evaluation shows a statistically significant correlation between the presence of an enhanced care team, and the improvement in scene times and MTDT compliance in the context of central stabbing. Within the dataset there also appears to be a considerable improvement in the number of patients who are triaged to a MTC when attended by an ECT. Both reduced scene times and triage compliance have been shown to be of benefit to patient outcomes [4, 21–23], so these findings are potentially of clinical significance.

The reasons for the results are likely to be multi-factorial. It has already been described how the ECTs in this study have a higher level of education compared to non-ECTs. It seems likely that this, coupled with an increased exposure to these incidents, will mean they are better prepared for the challenges which victims of penetrating trauma provide. They also have increased training in the subject of CRM, the importance of which is becoming increasingly recognised in pre-hospital and high acuity care [24, 25]. In this way, these results would seem to support the position that an important benefit which an ECT brings to the penetrating trauma patient is scene/team management; an understanding of the importance of shorter scene times, and the driving force to help achieve them. By the same token, the results also potentially highlight a training need amongst the non-ECT personnel, and an approach to improving their understanding of what is needed in such circumstances should be encouraged. It should be noted that, whilst ECTs undoubtedly have an increased frequency of exposure to central stabbing incidents, when compared to non-ECTs, the numbers involved are still in single figures per clinician, per year. This supports the premise that other factors, such as education level, robust governance systems and frequent training, are all important factors in the results seen.

That said, whilst the median total scene time was significantly shorter when ECTs attended, it is still higher than the generally accepted ideal of ten minutes [9]. This suggests there is still work to be done and improvements to be made. One explanation is the increased distance that these specialist units travel to get to scene, as well as the imperative of planning time etc. for helicopter response. A study of Texas-based HEMS units showed that not only scene times, but also response times can impact on mortality [10]. Certainly, this study has highlighted scope for improvement therefore, and more work needs to be done in investigating why the ten-minute goal remains challenging. If the reason is indeed the increased response times, then possible answers may include improving educational and real-time remote support for crews in deciding whether to set up rendez-vous points with ECTs en-route into an MTC.

As well as emphasising the importance of swiftness to the ECTs then, these results have potentially highlighted a training need amongst the non-ECT personnel, and an approach to improving their understanding of what is needed in such circumstances should be encouraged. This could take the form of both CPD in a structured/semi-structured learning environment, as well as increased emphasis on accessing remote support during the incident from the trust’s critical care desk.

### Limitations

This service evaluation was based on a small cohort, derived from retrospective data. This limits both the strength of conclusions which can be drawn, and the generalisability of the results, and is the reason why the aims of the study were correspondingly modest.

The two groups were unmatched, and it is unknown whether there was bias arising from disproportionate rates of confounding factors in either group.

It should be noted when looking at the average on scene times that our results carry with them several limitations. Police rendezvous arrangements, uncooperative patients, consent issues, and other confounding factors have all led to a wide variance in the results, with outliers still affecting the median to some degree.

It is also acknowledged that there are other interventions that form an entire treatment package, such as analgesia, tranexamic acid (TXA) and fluid therapy (in the context of permissive hypotension). These did not form part of this study, but should not be interpreted as being of little importance.

Other limiting factors must be taken in to account. As previously mentioned, some incidents may have been missed due to inaccurate categorisation in the call taking stage. The study does also not take in to account any advanced interventions that may have been needed at scene, prior to moving.

### Recommendations for future research

Scene delays are of proven importance in trauma, but in terms of measuring service effectiveness they are a proxy measure only. A prospective cohort study to calculate an adjusted odds ratio for mortality and morbidity (taking into account Injury Severity Score) could provide a powerful quantitative gauge of the ultimate goals of preserving life and facilitating as full a recovery as possible. Further studies should also include larger treatment packages, such as the administration of TXA. The inclusion of Injury Severity Score (ISS) details would be useful, though not routinely collected in the prehospital environment.

Qualitative research into the team dynamics in ECT led and non-ECT led teams could provide valuable insight into how the group dynamics differ, and how/why differences in on scene times come about.

A follow up analysis should also be undertaken, once the education lessons from such research have been disseminated. This will go some way to showing whether scene times and triage compliance, both with and without ECTs, has improved as a result.

## Conclusion

The results suggest that the presence of an enhanced care team has a beneficial effect on reducing scene times and on achieving appropriate patient triage of central penetrating trauma patients. Of the dataset studied, patients seen by ECTs were significantly more likely to be conveyed to a hospital sooner, and it was significantly more likely that that hospital would be a MTC., Further research is warranted to explore a possible positive patient outcome benefit tasking enhanced care teams to penetrating trauma may have.

## Data Availability

The original records used in this study are not publicly available and remain the property of South East Coast Ambulance Service NHS Foundation Trust. The processed data is available upon reasonable request from the corresponding author.
